# Commentary: Global, regional, and national burden of neurological disorders during 1990–2015: a systematic analysis for the Global Burden of Disease Study 2015

**DOI:** 10.3389/fneur.2018.00201

**Published:** 2018-03-29

**Authors:** Thomas K. Karikari, Augustina Charway-Felli, Kina Höglund, Kaj Blennow, Henrik Zetterberg

**Affiliations:** ^1^School of Life Sciences, Midlands Integrative Biosciences Training Partnership, University of Warwick, Coventry, United Kingdom; ^2^Neurology Department, 37 Military Hospital, Accra, Ghana; ^3^Department of Psychiatry and Neurochemistry, Institute of Neuroscience and Physiology, The Sahlgrenska Academy, The University of Gothenburg, Mölndal, Sweden; ^4^Clinical Neurochemistry Laboratory, Sahlgrenska University Hospital, Mölndal, Sweden; ^5^Department of Molecular Neuroscience, UCL Institute of Neurology, London, United Kingdom; ^6^UK Dementia Research Institute at UCL, London, United Kingdom

**Keywords:** biomarkers, Alzheimer’s disease, dementia, neurodegenerative diseases, low- and middle-income countries

Quality of life and longevity have increased in low- and middle-income countries (LMICs) but little is known about how increased aging may impact neurological health (1). In their recent *Lancet Neurology* paper, Feigin and colleagues (2) shed more light on this. By reanalyzing data from the 2015 Global Burden of Disease, the authors provided updated neurological disorder burden estimates. They found that Alzheimer’s disease (AD) and other dementias were the fourth-leading cause of deaths and disability globally, and consistently among the top three causes of disability in most countries (2).

Notably, the authors used proxy data from high-income countries to estimate dementia prevalence and mortality in LMICs due to data scarcity. This absence of epidemiological neurology data is partly due to a lack of screening tests and biomarker analysis adapted to local and social contexts for clinical decision making. In some settings, cognitive impairment assessment is the sole diagnostic criteria, and even this is not performed in many places (3). We believe that routine biomarker testing for patients with sufficient risk factors and screened for cognitive impairment would enhance clinical diagnosis and enrich epidemiological studies. For example, blood and cerebrospinal fluid (CSF) concentrations of amyloid-β, total tau, and phosphorylated tau robustly predict neurodegeneration progression (4). The cost-effectiveness of biomarker analyses favors their use in LMICs and may be particularly important in differentiating AD-like cognitive impairment from that of other causes (e.g., HIV), given their distinct CSF biomarker patterns (5).

Since cognitive assessment tools cannot efficiently distinguish between risk factors for AD (pathology), age-related dementia, and concomitant cerebrovascular disease (or other pathologies), biomarkers would be important in epidemiological studies aimed at revealing the true risk factors for these diseases. In addition to tau and amyloid-β, the emerging biomarkers neurofilament light chain, neurogranin, and YKL-40 sensitively predict early neurodegeneration, and age-related cognitive decline with or without neurodegeneration (6, 7). These biomarkers have great potential for identifying disease- and condition-specific risk factors in different environments.

A predictable challenge to the recommended approach is the lack of dedicated neurology diagnosis facilities. However, most LMICs have well-resourced public health laboratories that could be expanded to offer centralized biomarker testing services, with training and support from expert clinical biochemists and neurologists. These public health laboratories are equipped with molecular testing facilities and have been instrumental in managing emergencies such as the recent Zika and Ebola outbreaks. Governments should therefore prioritize biomarker-supported dementia diagnosis to enhance patient care, public health planning, and epidemiological studies, toward achieving the Sustainable Development Goals and related targets to defeat dementia.

## Author Contributions

All authors contributed to the preparation and editing of this manuscript.

## Conflict of Interest Statement

TK, AC-F, and KH have nothing to declare. KB and HZ are founders of Brain Biomarker Solutions in Gothenburg AB, a GU Ventures-based platform company at the University of Gothenburg. KB has served as a consultant or at advisory boards for Fujirebio Europe, IBL International, and Roche Diagnostics, outside the submitted work. HZ has received travel support from Teva and has served at advisory boards for Roche Diagnostics and Eli Lilly, outside of the submitted work.
